# Comparing Four Video Laryngoscopes and One Optical Laryngoscope with a Standard Macintosh Blade in a Simulated Trapped Car Accident Victim

**DOI:** 10.1155/2019/9690839

**Published:** 2019-10-01

**Authors:** Florian J. Raimann, Daniel M. Tepperis, Dirk Meininger, Kai Zacharowski, Richard Schalk, Christian Byhahn, Christian F. Weber, Haitham Mutlak

**Affiliations:** ^1^Department of Anesthesiology, Intensive Care Medicine and Pain Therapy, University Hospital Frankfurt, Goethe University, Frankfurt, Germany; ^2^Main-Kinzig-Clinic, Department of Anesthesia, Intensive Care Medicine and Pain Therapy, Herzbachweg 14, 63571 Gelnhausen, Germany; ^3^Evangelical Hospital Oldenburg, Department of Anesthesia, Intensive Care Medicine and Pain Therapy, Steinweg 13-17, 26122 Oldenburg, Germany; ^4^Asklepios Clinics Hamburg, Asklepios Clinic Wandsbek, Department of Anesthesiology, Intensive Care Medicine and Emergency Medicine, Alphonsstraße 14, 22043 Hamburg, Germany

## Abstract

**Background:**

Tracheal intubation still represents the “gold standard” in securing the airway of unconscious patients in the prehospital setting. Especially in cases of restricted access to the patient, video laryngoscopy became more and more relevant.

**Objectives:**

The aim of the study was to evaluate the performance and intubation success of four different video laryngoscopes, one optical laryngoscope, and a Macintosh blade while intubating from two different positions in a mannequin trial with difficult access to the patient.

**Methods:**

A mannequin with a cervical collar was placed on the driver's seat. Intubation was performed with six different laryngoscopes either through the driver's window or from the backseat. Success, C/L score, time to best view (TTBV), time to intubation (TTI), and number of attempts were measured. All participants were asked to rate their favored device.

**Results:**

Forty-two physicians participated. 100% of all intubations performed from the backseat were successful. Intubation success through the driver's window was less successful. Only with the Airtraq® optical laryngoscope, 100% success was achieved. Best visualization (window C/L 2a; backseat C/L 2a) and shortest TTBV (window 4.7 s; backseat 4.1 s) were obtained when using the D-Blade video laryngoscope, but this was not associated with a higher success through the driver's window. Fastest TTI was achieved through the window (14.2 s) when using the C-MAC video laryngoscope and from the backseat (7.3 s) when using a Macintosh blade.

**Conclusions:**

Video laryngoscopy revealed better results in visualization but was not associated with a higher success. Success depended on the approach and familiarity with the device. We believe that video laryngoscopy is suitable for securing airways in trapped accident victims. The decision for an optimal device is complicated and should be based upon experience and regular training with the device.

## 1. Introduction

Tracheal intubation still represents the most common way in securing the airway of unconscious patients in the prehospital setting and remains therefore an important clinical skill for emergency physicians [1]. Tracheal intubation failure is associated with an increase in mortality and morbidity. For example, esophageal intubation, which occurs in up to 25% in prehospital airway management, increases the 24 h mortality from 10% to 70–90% in a prehospital emergency collective [2].

Video laryngoscopy has become a powerful aid in this aspect and has shown to improve the management of expected and unexpected difficult airway intubation in the clinical setting and especially for training purposes in the academic setting [3–5]. Video laryngoscopy has been implemented in many institutions as part of the airway management standard operating procedures, in addition to being in the international airway management guidelines [6, 7]; within the last decade, various video laryngoscopes by different manufacturers have flooded the market, thus making it difficult to choose the optimal device for different scenarios and circumstances.

Airway management in the prehospital setting is generally more challenging. Under certain circumstances, the access to the patient is limited, and therefore, intubation conditions are even more difficult than normal ones [8]. Another important aspect is the increased risk of aspiration based on nonfasting patients and, if cervical spine trauma is suspected, the need for cervical immobilization during the intubation process. Immobilization of the cervical spine complicates the intubation procedures in a time-critical environment. It has been shown that there are a significantly higher incidence of difficult airway [9] and an increased mortality risk within this subset of patients [10], especially in major trauma patients associated with a coexisting cranial pathology (e.g., Glasgow coma scale score of 8 or less). These patients have the highest aspiration risk and are in critical need of oxygen supply; hence, securing the airway of these patients is of utmost urgency [8, 11].

Tracheal intubation is a skill which needs to be trained regularly [12]. Even with an adequate view of the glottis, tracheal intubation can be challenging with different intubation success rates depending on the provider's experience [13, 14]. However, the low frequencies of prehospital intubations are a hindrance to gaining adequate or routine experience with the procedure.

The regular use of video laryngoscopes in the (pre)hospital setting has shown benefits in respect of the optimized view of the glottis and in intubation success rates [15–17].

The aim of the present study was to evaluate the performance and intubation success rates of four different video laryngoscopes and one optical scope comparing with the direct laryngoscopy using a standard Macintosh blade while intubating from two different positions. Furthermore, we compared time to intubation, time to best view, and failed intubations in a scenario of a trapped car accident victim with an airway mannequin.

## 2. Materials and Methods

### 2.1. Study Design

The study was designed as a prospective mannequin study. Since all investigations were carried out on mannequins, no approval of the local ethics committee was required.

**Figure 1:**
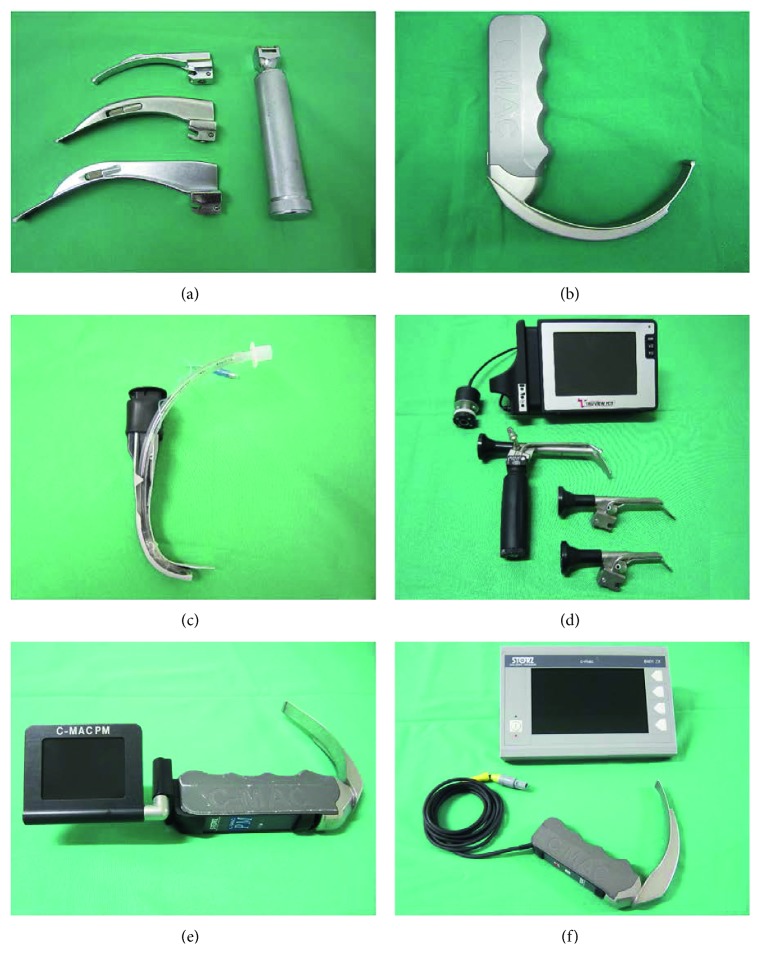
Intubation devices. (a) Macintosh blade sizes 2, 3 and 4. (b) D-Blade. (c) Airtraq® with a tube. (d) Truview PCD™-R with different blade sizes. (e) C-MAC® PM. (f) C-MAC® with an external TFT monitor.

All participants had to perform tracheal intubation with six different devices using two different approaches to the patient:

- Standard laryngoscope (KaWe-Kirchner & Wilhelm GmbH & Co. KG, Asperg, Germany) with a size 3 Macintosh blade (Figure 1(a))
- Storz D-Blade + standard external monitor (Karl Storz GmbH & Co. KG, Tuttlingen, Germany) (Figure 1(b))
- Airtraq® optical laryngoscope of size 3 (Prodol Mediteq, S.A., Madrid, Spain), which has two anatomical shaped channels for optic guidance and for guidance of the tracheal tube (Figure 1(c))
- Truview PCD™-R EVO2 with a size 3 blade and standard external monitor (Truphatek, Netanya, Israel) (Figure 1(d))
- Storz C-MAC® Pocket Monitor (PM) with a size 3 blade (Karl Storz GmbH & Co. KG, Tuttlingen, Germany) (Figure 1(e))
- Storz C-MAC® with a size 3 blade and standard external monitor (Karl Storz GmbH & Co. KG, Tuttlingen, Germany) (Figure 1(f))

To ensure adequate handling with the designed devices, all participants had to perform a minimum of 5 intubations with each device. Therefore, an airway management training was performed two months in advance of the planned investigation using an intubation mannequin (Ambu® Airway Man I, Ambu GmbH, Bad Nauheim, Germany).

### 2.2. Participants

All participants were staff of the university hospital where the study was conducted. According to national guidelines [12], emergency physicians are declared as experienced, if more than 100 initial intubations and at least 10 intubations/year are completed. 95.2% (*n* = 40) of all participants met this criterion. Subgroup analysis was performed according to the professional experience. Therefore, the participants were divided into two groups. Group one was represented by interns (*n* = 22; <5 years of experience), and the second group was board-certified physicians (*n* = 20; >5 years of experience).

### 2.3. Study Setting

**Figure 2:**
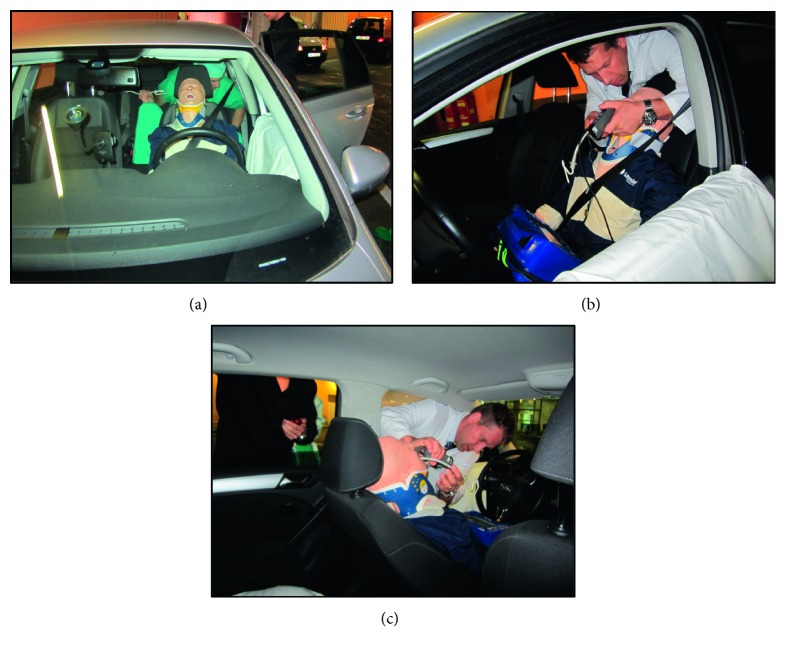
Setting. (a) Position of the mannequin in the car with a fasted seatbelt, upright position, and cervical collar. (b) Position for tracheal intubation from the backseat. (c) Position for tracheal intubation from the driver's window.

We created a scenario in which the emergency physician was confronted with a scene, where immediate emergency intubation was necessary. Therefore, we simulated a major trauma scene with a trapped person on the driver's seat. A mannequin (MegaCode Kelly™ Advanced, Laerdal Medical, Stavanger, Norway) was placed on the driver's seat of a VW Golf VI car (VW Group, Wolfsburg, Germany). The seat belt was fastened around the torso of the mannequin, and a cervical collar (Stifneck® Select™ Adult, Laerdal Medical, Stavanger, Norway) was placed (Figure 2(a). Reclination of the driver's seat, opening the side door, or removal of the cervical collar for intubation was not allowed. All six devices were used in two different approaches to secure the airway of the mannequin. One intubation approach was through the driver's window, and the second approach was from the backseat of the car (Figures 2(b) and 2(c)). There was no assistance of a second person.

### 2.4. Interventions

It was the participants' choice to start either through the driver's window or from the backseat. The sequence of the six intubation devices was randomized at the beginning of the experiment. A prepared and lubricated Magill tracheal tube with 7.5 mm internal diameter, a 10 ml syringe to inflate the cuff, and a self-inflating Ambu bag (Ambu Spur I®, Ambu GmbH, Bad Nauheim, Germany) were supplied, except for the intubation with the Airtraq optical laryngoscope where a preformed intubation stylet was introduced into the tracheal tube. Correct placement was checked after intubation by one of the investigators by examining the mannequin. A questionnaire asking for qualification, years of medical experience, qualification as an emergency physician, experience with the supplied devices, and private use of gaming consoles (supplemental data) had to be filled out by every participant.

### 2.5. Outcome Measures

The primary endpoint is the successful tracheal intubation.

The secondary endpoints are as follows:

- Time to best view (TTBV)
- Time to intubation (TTI)
- Visualization of the glottis graded by the Cormack–Lehane scale [18, 19]
- Correct placement of a tracheal tube
- Favored device

### 2.6. Data Analysis

Analysis of the data confirmed that no Gaussian distribution was present. All data were presented as median and 25th/75th percentile. Statistical analysis was carried out using the software package GraphPad Prism (Version 5.01, GraphPad Software Inc., San Diego, CA). Data were analyzed with the nonparametric Kruskal–Wallis one-way analysis of variance (ANOVA) test, and post hoc analysis was performed using Dunn's multiple-comparison test. Statistical significance was assumed with a probability of type I error less than 5% (*p* < 0.05).

## 3. Results

Forty-two physicians participated. Demographic characteristics and experience are shown in Table 1.

### 3.1. Success Rate

The primary endpoint of this investigation was the rate of successful intubation. Successful intubation rates and statistic comparison are shown in Table 2. The success rate from the backseat was 100%. A 100% success rate when intubation was performed through the window was achieved only with the Airtraq® device. The lowest intubation rate through the window was accomplished with Truview PCD™-R (74%). There was no difference detected in a subgroup analysis between interns (<5 years of experience) and board-certified physicians (>5 years of experience). The results are depicted in [Supplementary-material supplementary-material-1] (online supplemental material).

### 3.2. Glottis Visualization

**Figure 3:**
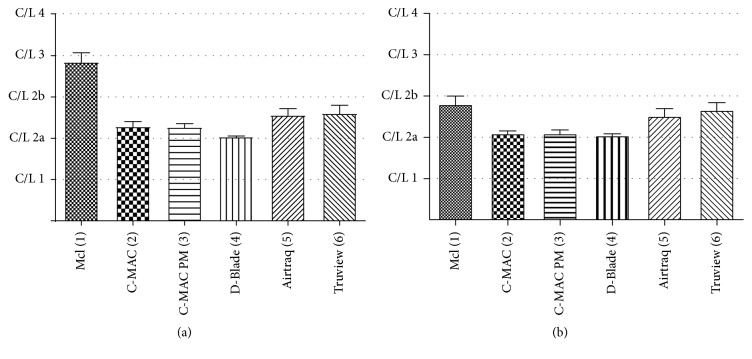
C/L classification. (a) Rating from the window approach. McI = Macintosh (direct laryngoscopy). Devices 2, 3, 4, 5, and 6 show significant results. (b) Rating from the backseat approach. Devices 2, 3, and 4 show significant results.

In Figure 3, visualization of the glottis is displayed according to the intubation approach (Figures 3(a) and 3(b)). Performing direct laryngoscopy with a Macintosh blade while approaching through the window revealed a significantly inferior visualization (C/L 3) compared to all other indirect laryngoscopy devices. When intubation was performed via a backseat approach, the direct laryngoscopy with a Macintosh blade revealed an improved visualization. However, the aforementioned visualization was significantly inferior to an indirect visualization with C-MAC®, C-MAC® PM, and D-Blade. Additionally, Truview PCD™-R showed a significant inferior visualization of the glottis compared to D-Blade (Figure 3(b)).

### 3.3. Time to Best View

Time to best view (TTBV) was reached faster from the backseat than through the window except when using the Airtraq® device (9.3 vs. 9.4 seconds). TTBV in direct laryngoscopy via a Macintosh blade was nearly halved when approaching from the backseat. Significances and values in seconds are shown in Table 3. No difference was detected between interns and board-certified physicians ([Supplementary-material supplementary-material-1]).

### 3.4. Time to Intubation

Likewise, time to intubation (TTI) was faster when using the backseat approach, regardless of which device was used. The Airtraq® device showed the least difference in TTI in both approaches. Values in seconds and reached levels of significance are depicted in Table 3. No difference was detected between interns and board-certified physicians ([Supplementary-material supplementary-material-1]).

### 3.5. Laryngoscopy

The number of intubation attempts through the window was significantly higher compared to that of all other devices (C-MAC®, *p* < 0.001; C-MAC® PM, *p*=0.03; D-Blade, *p*=0.02; Airtraq®, *p*=0.008) with the exception of the Macintosh blade when using Truview PCD™-R (up to 4 attempts per participant).

When approaching from the backseat, significantly more attempts were performed with Airtraq® in comparison with the Macintosh blade (*p*=0.01) C-MAC® (*p*=0.01), and C-MAC® PM (*p*=0.02).

### 3.6. Failed Attempts and Esophageal Intubations

Based on all performed intubations, whether through the window or from the backseat, no undetected esophageal intubation was documented. Only with the Truview PCD™-R device compared to C-MAC® (*p*=0.02) and Airtraq® (*p*=0.02), the level of significance was reached when intubating through the window. When approaching from the backseat, Truview PCD™-R showed significantly poorer results compared to the Macintosh blade (*p* < 0.001), C-MAC® (*p* < 0.001), and C-MAC® PM (*p*=0.005).

In regard to the esophageal intubations, all esophageal intubations were recognized by the participants and corrected within 120 seconds.

Only with the Truview PCD™-R device compared to C-MAC® (*p* < 0.001) and Airtraq® (*p* < 0.001), the level of significance was reached when intubating through the window. When approaching from the backseat, Truview PCD™-R showed significantly worse results compared to the Macintosh blade (*p*=0.012), C-MAC® (*p* < 0.012), and C-MAC® PM (*p*=0.018).

### 3.7. Favored Devices

C-MAC® was the most favored device by participants (*n* = 20; 48%). Analyzing interns and board-certified anesthesiologists revealed differences in their favorites. Besides C-MAC®, which reached number one place in both subgroups, board-certified anesthesiologists preferred the direct laryngoscopy method. Interns voted for C-MAC® PM for the second place. The ranking for subgroups and overall ranking are shown in Table 4.

## 4. Discussion

### 4.1. Interpretation of Findings

The aim of the present study was to compare four different video laryngoscopes, one optical laryngoscope, and a standard Macintosh blade in a simulated difficult airway in a trapped car accident victim regarding intubation success. The main finding of the present study was that the highest overall intubation success was achieved when approaching from the backseat, regardless of the laryngoscope used. When intubating through the driver's window, the success rate varied between 74% and 100%, depending on the device (Table 2). The underlying reasons for these varying success rates may have been a more difficult access to the patient, and the need of an “upside-down” intubation (Figure 2(c)) [20, 21].

### 4.2. Comparison to Previous Studies

Wetsch et al. reported a success rate of 88% in a similar mannequin study. Here, our results were confirmed by intubation from the driver's side with a standard Macintosh laryngoscope, C-MAC, Airtraq, Glidescope Ranger, Ambu Pentax, and McGrath [22]. Timmermann et al. described 6.1% failed intubations when intubation was performed by physicians in emergency situations; however, a different access to the patient was used [21]. When intubation was performed by paramedics, the rate of failed intubations increased up to 31% [23].

Successful intubation is strongly affected by visualization of the glottic structures. Achieving quickly a optimal view, described as the time to best view (TTBV), has impact on the total duration of the intubation process and has therefore influence on the time to intubation (TTI). It has been shown that visualization of the glottis was improved with the utilization of video laryngoscopes in various clinical settings [15–17, 24]. In our study, the fastest TTBV was achieved with D-Blade, independent of the approach. D-Blade was invented especially for patients immobilized by a cervical collar, and therefore, the high angulated shape of this blade may be responsible for the fastest time to best view [25]. When performing the intubation from the backseat, TTBV using the Airtraq device was the longest. This can be explained by the fact that eye placement directly on the ocular from the backseat is challenging with this optical laryngoscope. However, also with the backseat approach, a 100% intubation success rate was observed. In contrast to the other video laryngoscopes, the eye-hand coordination is affected by the tube guide channel, leading to a simplified insertion of the tube, once the best view via the ocular is achieved. This rather allows inexperienced users a successful intubation [26–28].

However, in the present investigation, optimized visualization was not associated with a higher intubation success rate [29–31]. It has been demonstrated in previous studies that the sharply angled blade was detected as the main cause of prolonged and failed intubations [29, 32]. In our study, better visualization did not correlate with a higher rate of successful intubations independently of the used laryngoscope, which has been demonstrated before by various groups [29–31].

It turned out that the fastest TTBV with the most devices was reached when accessing the mannequin from the backseat. This may be explained by a more familiar access to the mannequins' head (Figure 2(c)). Regardless of the participant's approach, direct laryngoscopy with a Macintosh blade revealed the poorest visualization and was significantly improved when VLs were used (Figure 3).

TTI from the backseat was shorter compared with that of the approach through the window, which could be explained by a different and uncommon access to the patients' airway. Interestingly, the fastest TTI from the backseat was achieved when using the Macintosh blade for direct laryngoscopy, despite the fact that visualization was worse when using this device compared to the video laryngoscopes. A possible explanation for this result may be the highest level of experience with this device. Noppens et al. published comparable results in the ICU setting. Experienced Macintosh blade users were able to secure a difficult airway with a standard Macintosh blade after failed intubation using a video laryngoscope [17]. Another reason may be different angulations of the other blades. Some devices require a special guide rod or preformed stylet [29]. Proper handling and eye-hand-tube coordination [33] during indirect laryngoscopy could only be performed by a trained person. Therefore, regular training is important for successful handling [2, 34].

Although fastest intubation was performed using a standard Macintosh blade, C-MAC® with a Macintosh blade was the users' favored device. This may be related to our standard operating procedure when using this VL system in expected and unexpected difficult airway algorithms, therefore resulting in a familiarity with the device.

The results of the present study show that the practitioner's level of experience with a particular device has a greater impact on the success of intubation than the type of the equipment. Although the use of video laryngoscopes has increased significantly in recent years, traditional laryngoscopes are still a frequently used device and still guarantee a high success rate of intubation.

In many countries, tracheal intubation in the preclinical setting is performed by EMTs (emergency medical technicians), which often have little experience in tracheal intubation, neither with video laryngoscopes nor with traditional laryngoscopes. Here, alternative airway devices like video laryngoscopes provide a good alternative for the inexperienced user [35, 36].

### 4.3. Strengths and Limitations

Our study has methodological limitations. We aimed to simulate a realistic scenario using a mannequin, although former studies showed that mannequins differ from human airway anatomy [37]. Results obtained in simulated scenarios using mannequins should therefore be interpreted with caution. Nevertheless, mannequins are often used for the evaluation of intubation devices in simulated trapped accidents [22, 38]. This is due to the fact that situations with a difficult access to the patient with the necessity to secure the airways are rare, therefore affecting the study duration. Furthermore, sequential intubation with different devices under difficult intubation conditions in humans was impossible for ethical reasons. Another important limitation was the unequal experience of the participants regarding the investigated devices. Although practical training in advance of the study was performed, the experience with the C-MAC system was higher and may have an influence on the results. Besides these limitations, different levels of experience may influence the results. Performing subgroup analysis revealed no significant differences between interns and board-certified physicians. Nevertheless, a bias cannot be ruled out.

At last, we compared one optical device with four video laryngoscopes. Different handling and restricted access to the device based on limited access to the mannequin may have influenced our results.

## 5. Conclusion

The results of the present investigation showed that video laryngoscopes could be an appropriate alternative for prehospital emergency intubation in patients with a difficult access to the airways. We demonstrated that VL improves the view of the glottic structures, but good view was not associated with a successful intubation depending on the angulation of the blade. Direct laryngoscopy with a standard Macintosh blade may be a rescue option, especially in experienced users.

Regular training and clinical experience must be assured in the use of VL devices, regardless of the type of VL used. As the intubation procedure from the backseat was associated with faster TTBV and TTI, this position should be favored, if possible.

## Figures and Tables

**Table 1:** Demographics, experience, and qualification of the participants (*n* = 42).

Participants	42	100%
Female	13	31%
Male	29	69%

Qualification		
Anaesthesiology interns	21	50%
Board-certified anesthesiologists	19	45%
Interns with different specialization	2	5%

Additional qualification		
Emergency physicians	30	71%

Experience with used devices		
Macintosh blade	40	95%
C-MAC®	40	95%
C-MAC® PM^*∗*^	0	0%
D-Blade	14	14%
Airtraq® SP	10	24%
Truview PCD™-R	16	38%

Experience as physician (overall)	6.0	3.0/9.0
Anesthesiology interns	4.5	2.0/5.0
Board-certified anesthesiologists	9.0	6.5/11.5
Interns with different specialization	3.5	3.3/3.8
Experience as emergency physician	3.8	2.8/6.0

^*∗*^Introduced in our department directly before the investigation.

**Table 2:** Success rate of intubation from the window and backseat positions.

		McI (1)	C-MAC® (2)	C-MAC® PM (3)	D-Blade (4)	Airtraq® SP (5)	Truview PCD™-R (6)
Window	*n* (%)	41/42 (98)	39/41 (95)	34/36 (94)	36/42 (86)	42/42 (100)	29/39 (74)
	Sign.	NS	NS	NS	NS	NS	1, *p*=0.003^*∗*^
							2, *p*=0.02^*∗*^
							3, *p*=0.03^*∗*^
							5, *p* < 0.001^*∗*^

Backseat	*n* (%)	42/42 (100)	41/41 (100)	36/36 (100)	42/42 (100)	42/42 (100)	39/39 (100)
	Sign.	NS	NS	NS	NS	NS	NS

McI = Macintosh (direct laryngoscopy); NS = nonsignificant; ^*∗*^significant with less intubation success in comparison with all other devices except D-Blade.

**Table 3:** Time to best view (TTBV) and time to intubation (TTI).

			McI (1)	C-MAC® (2)	C-MAC® PM (3)	D-Blade (4)	Airtraq® SP (5)	Truview PCD™-R (6)
Time to best view (TTBV)	Window	Median (25th/75th percentile) (s)	10.4 (5.7/14.9)	6.0 (3.8/8.6)	8.0 (4.8/13.4)	4.7 (3.3/7.8)	9.3 (6.9/15.4)	10.1 (5.8/23.1)
		Sign.	2, *p*=0.05^*∗*^ 4, *p* < 0.001^*∗*^	5, *p*=0.008^*∗*^ 6, *p*=0.01^*∗*^	NS	5, *p* < 0.001^*∗*^ 6, *p* < 0.001^*∗*^	NS	NS
	Backseat	Median (25th/75th percentile) (s)	4.8 (3.5/5.8)	4.4 (3.6/5.5)	5.1 (3.6/6.8)	4.1 (3.0/5.8)	9.4 (6.3/15.9)	7.1 (4.2/11.1)
		Sign.	5, *p* < 0.001^*∗*^ 6, *p*=0.04^*∗*^	5, *p* < 0.001^*∗*^ 6, *p*=0.004^*∗*^	5, *p* < 0.001^*∗*^	5, *p* < 0.001^*∗*^ 6, *p*=0.001^*∗*^	NS	NS

Time to intubation (TTI)	Window	Median (25th/75th percentile) (s)	21.1 (14.7/39.0)	14.2 (9.7/23.2)	20.9 (15.0/31.8)	20.9 (14.1/67.2)	16.7 (11.8/34.0)	37.4 (18.4/117.6)
		Sign.	NS	6, *p* < 0.001^*∗*^	NS	NS	6, *p*=0.008^*∗*^	NS
	Backseat	Median (25th/75th percentile) (s)	7.3 (5.9/9.5)	8.8 (6.9/10.3)	9.5 (7.2/13.8)	12.6 (8.6/18.9)	14.6 (10.3/27.1)	17.7 (11.8/47.1)
		Sign.	4, *p* < 0.001^*∗*^ 5, *p* < 0.001^*∗*^ 6, *p* < 0.001^*∗*^	4, *p*=0.005^*∗*^ 5, *p* < 0.001^*∗*^ 6, *p* < 0.001^*∗*^	5, *p*=0.004^*∗*^ 6, *p* < 0.001^*∗*^	NS	NS	NS

McI = Macintosh (direct laryngoscopy); NS = nonsignificant; ^*∗*^significant; s = seconds; lower median indicates faster TTBV or TTI.

**Table 4:** Evaluation of preferred devices after examination.

Experience	Placement	Device	*n*	Percent
Overall	1	C-MAC®	20	48
	2	C-MAC® PM	8	19
	3	Macintosh blade	6	14
	4	D-Blade	6	14
	5	Airtraq® SP	2	5
	6	Truview PCD™-R	0	0

Board-certified physicians	1	C-MAC®	8	42
	2	Macintosh blade	5	26
	3	D-Blade	4	21
	4	C-MAC® PM	2	11
	5	Airtraq® SP	0	0
	6	Truview PCD™-R	0	0

Interns	1	C-MAC®	12	55
	2	C-MAC® PM	5	23
	3	D-Blade	2	9
	4	Airtraq® SP	2	9
	5	Macintosh blade	1	5
	6	Truview PCD™-R	0	0

## Data Availability

Supporting data can be obtained from the corresponding author upon request.

## Supplementary Materials

Supplementary Materials Comparison between interns and board-certified physicians.
