# Supplementary material for: Comparing Four Video Laryngoscopes and One Optical Laryngoscope with a Standard Macintosh Blade in a Simulated Trapped Car Accident Victim
Source: Emerg Med Int. 2019 Oct 1;2019:9690839. doi: 10.1155/2019/9690839 (PMC6791209; doi:10.1155/2019/9690839)
Supplement: Supplementary Materials — Comparison between interns and board-certified physicians. [file 9690839.f1.pdf]

Table 5 Comparison between interns and board-certified physicians.

			McI (1)	C-MAC® (2)	C-MAC® PM (3)	D-Blade (4)	Airtraq® SP (5)	Truview PCD™-R (6)
Interns vs. board-certified	Window	Success	ns	ns	ns	ns	ns	ns
		TTBV	ns	ns	ns	ns	ns	ns
		TTI	ns	ns	ns	ns	ns	ns
	Backseat	Success	ns	ns	ns	ns	ns	ns
		TTBV	ns	ns	ns	ns	ns	ns
		TTI	ns	ns	ns	ns	ns	Ns

McI = Macintosh (direct laryngoscopy); ns = non-significant; * = significant
Level of significance was not reached in any comparison between interns and board-certified physicians.
